# Manipulation of Intensive Longitudinal Data: A Tutorial in R With Applications on the Job Demand‐Control Model

**DOI:** 10.1002/ijop.70040

**Published:** 2025-03-23

**Authors:** Luca Menghini, Enrico Perinelli, Cristian Balducci

**Affiliations:** ^1^ Department of General Psychology University of Padova Padua Italy; ^2^ Department of Psychology and Cognitive Science University of Trento Trento Italy; ^3^ Department of Psychology University ‘G. d'Annunzio' of Chieti‐Pescara Chieti Italy

**Keywords:** data pre‐processing, intensive longitudinal designs, introductory guidelines, job demand‐control model, multilevel modelling

## Abstract

Intensive longitudinal designs (ILD) are increasingly used in applied psychology to investigate research questions and deliver interventions at both within‐ and between‐individual levels. However, while relatively complex analyses such as cross‐level interaction models are trending in the field, little guidance has been provided on ILD data manipulation, including all procedures to be applied to the raw data points for getting the final dataset to be analysed. Here, we provide an introductory step‐by‐step tutorial and open‐source R code on *required* and *recommended* data pre‐processing (e.g., data reading, merging and cleaning), psychometric (e.g., level‐specific reliability), and other ILD data manipulation procedures (e.g., data centering, lagging and leading). We built our tutorial on an illustrative example aimed at testing the job demand‐control model at the within‐individual level based on data from 211 back‐office workers who received up to 18 surveys over three workdays, supporting both the strain and (partially) the buffer hypotheses. Being the common starting point of many types of analyses, data manipulation is crucial to determine the quality and validity of the resulting study outcomes. Hence, this tutorial and the attached code aim to contribute to removing methodological barriers among applied psychology researchers and practitioners in the handling of ILD data.

## 1 Introduction

The occurrence of change over time in cognitions and behaviours is a key assumption that virtually applies to any psychological theory and intervention, making the study of temporal dynamics particularly critical for the advancement of applied psychology. Starting from the seminal book by Nesselroade and Baltes (1979), longitudinal designs ranging from panel studies to intensive longitudinal designs (ILD) have become widely used in behavioural science. Particularly, ILD is an umbrella term for multiple data sampling techniques that typically involve numerous data points (e.g., 10‐to‐50 observations per participant) and short time lags (e.g., minutes, days, weeks) to capture temporal dynamics that cannot be unravelled with lower sampling rates (Bolger and Laurenceau 2013; Revelle and Wilt 2019).

ILD such as daily diaries and experience sampling methods (ESMs) are increasingly used in applied psychology to test the conceptualisation of multilevel constructs such as work‐related stressors and moods (e.g., Menghini et al. 2023), track psychophysiological dynamics conditional on work characteristics (e.g., Baethge et al. 2020) and evaluate the homology of nomological networks across the within‐ and between‐individual levels (Gabriel et al. 2019). Indeed, ILD can be used to draw insights on both (a) intra‐individual fluctuations over time (level 1: within) and (b) inter‐individual differences (level 2: between), as well as on (c) cross‐level interactions. For instance, Balducci et al. (2024) found that time‐invariant variables (e.g., traumatic event) can moderate the intra‐individual relationships between time‐varying measures of stressors (e.g., exposure to bullying) and strains (e.g., post‐traumatic stress symptoms). Moreover, ILD such as ecological momentary assessments and interventions are of increasing interest for the practitioner community as well (Balaskas et al. 2021).

Critically, the ability of ILD to capture short‐term changes comes at the cost of greater dataset complexity, requiring specific procedures (e.g., handling missing observations, merging multiple datasets) to ensure acceptable data quality. Yet, despite the increasing use of complex statistical techniques to analyse ILD datasets, little guidance has been provided so far on the preliminary steps that should be undertaken to evaluate ILD data and prepare it for statistical analysis (Revol et al. 2024; Weermeijer et al. 2022). Data manipulation involves all procedures transforming the raw data functionally to, and compliant with the assumption of, the target statistical models. Although often perceived as a marginal aspect of data analysis, data pre‐processing has fundamental implications for the results of a study, especially with complex datasets such as in ILD. Moving from long‐ to wide‐form datasets, decomposing variables across levels and computing level‐specific psychometrics are examples of challenging procedures to process and evaluate data collected through ILD. Moreover, dataset complexity enlarges the sequence of procedures where one can arbitrarily choose among multiple forking paths, strongly impacting the study's conclusions (Weermeijer et al. 2022).

### 1.1 The Present Study

Here, we aim at providing introductory guidance to handle the complexity and challenges implied by ILD data manipulation, particularly for applied psychologists encountering ILD for the first time. To reach this aim, we propose a user‐friendly tutorial on how to prepare ILD raw data for most types of multilevel analyses.

Specifically, we present our recommendations based on an illustrative example considering time‐varying indicators of job task‐related demands, control and mood to test the job demand‐control (JDC) model at the within‐individual level. The JDC (Karasek 1979) is a popular model predicting worse strain reactions for job positions implying higher demands and lower control (*strain hypothesis*, i.e., focused on main effects), with the latter attenuating the negative effects of the former (*buffer hypothesis*, i.e., focused on interaction). So far, the JDC model has been mainly investigated at the between‐individual level. Only recently, some ILD studies used multilevel modelling to analyse repeated measures of ongoing demands and control and extend the JDC model at the daily or weekly level, although results have been mixed (Johnston et al. 2016; Totterdell et al. 2006). Thus, we aim to test the substantive hypotheses that daily fluctuations in mood levels are predicted by within‐individual fluctuations in task demand, task control and their interaction.

Building from this illustrative example, we aim to provide step‐by‐step guidance over the main procedures *required* (i.e., necessary to draw meaningful results) or *recommended* (i.e., suggested as best practices) for ILD data manipulation. The tutorial is designed to be easily accessible and flexibly applicable to virtually any kind of ILD data. Moreover, each step is accompanied by open‐source R code to be used by applied psychologists with varying levels of expertise. Following a description of the illustrative example, we introduce the rationale and the key procedures involved in each step, moving from data reading to the computation and transformation of composite scores. Then, we report multilevel analyses testing JDC hypotheses at the within‐individual level based on the processed data. Finally, we summarise and discuss the main challenges posed by ILD datasets and some potential solutions to improve the transparency and reproducibility of ILD data manipulation. The R code matching the step‐by‐step procedure outlined below is available at https://osf.io/sxh4q/.

## 2 Methods

### 2.1 Participants and Procedure

The illustrative example is based on the data collected by Menghini et al. (2023) from 211 back‐office workers^1^ (102 women, mean age = 35.81, SD = 9.93 years) employed in various sectors and positions (34.01% business and administration professionals/associate professionals, 18.01% science/engineering professionals, 47.58% other). The full description of participant recruitment and data collection is provided by Menghini et al. (2023), and the dataset was made publicly available at https://osf.io/87A9P/. Briefly, after filling out a preliminary questionnaire on demographic and other time‐invariant variables, participants used a mobile app to respond to ESM questionnaires at predefined times over three workdays (i.e., Monday, Wednesday and Friday)^2^. ESM questionnaires were scheduled every 90 ± 10 min randomly determined from 10:30 AM to 6:15 PM, resulting in up to six daily measurements.

### 2.2 Measures

Task demands were measured with four items adapted from the *Quantitative Workload Inventory* (Spector and Jex 1998) (e.g., “*I needed to work very hard*”), whereas task control was measured with three items from the *Diary for Ambulatory Behavioural States* (Kamarck et al. 2002) (e.g., “*I could change task if I chose to*”), being both referred to the job tasks performed over the last 10 min and responded from 1 = “*Not at all*” to 7 = “*Very much*”. Negative affective valence was measured with three bipolar items from the *Multidimensional Mood Questionnaires* (Wilhelm and Schoebi 2007) (e.g., “*How are you feeling right now?*”; 1 = “*Very well*”, 7 = “*Very unwell*”).

### 2.3 Step‐By‐Step Tutorial on ILD Data Manipulation

#### 2.3.1 Step 1. Data Reading

ILD data format can vary depending on the software/platform used to collect the data. Some recording systems automatically merge all measurements in a single ready‐to‐use dataset, whereas other systems export one or multiple files per participant, requiring post hoc data merging procedures. In any case, it is *recommended* to identify the main data columns of interest and remove unused columns. The variable identifying the rows associated with the same participant is particularly crucial since it allows grouping all data points corresponding to the same person, and it is used in subsequent steps for merging multiple data sources (Step 4) and decomposing the variance for psychometric (Step 6), descriptive and regression analyses. In our case, such variable was named ‘ID’ and retained as the first dataset column (see Table 1). Other critical data columns include the response temporal coordinates (date and time of response initiation/submission) and the item scores (here, the responses to task demands, task control and negative valence items). As these key variables are identified, they might be relabeled to optimise data manipulation. Indeed, strategically naming the variables to mark information such as reversed item scores or variable names is critical to prevent mistakes in the following steps and improve the effectiveness and transparency of the data analysis scripts.

**TABLE 1:** Output of temporal synchronisation.

ID	Time	Day	Beep	v1
S001	03/12/2018 10.22	1	2	4
S001	03/12/2018 12.05	1	3	3
S001	03/12/2018 13.27	1	4	2
S001	03/12/2018 18.00	1	7	2
S001	05/12/2018 11.56	2	3	3
S001	—	—	—	—
S001	25/01/2019 12.00	3	3	2
S001	—	—	—	—
S002	18/01/2019 09.15	1	1	3

*Note:* The table shows example rows for variables ‘ID’ (participant identifier), ‘time’ (response temporal coordinates), ‘day’ (day of participation), ‘beep’ (measurement occasion within ‘day’) and ‘v1’ (negative valence‐item 1).

#### 2.3.2 Step 2. Temporal Synchronisation

A *required* step functional to verify the correctness of data merging in Steps 1 (when multiple files are exported from the data collection system) and 4 (when multiple data sources are used) consists of ensuring that the temporal coordinates (date and time) associated with each data point are correctly recorded. For instance, this can be achieved by synching temporal variables with a common format and time zone, such as those of the local machine used to process the data. Furthermore, it is *recommended* to transform temporal coordinates into data point identifiers, that is, counter variables for the day of participation (here, named ‘day’) and the measurement occasion within each day (here, named ‘beep’), as both variables might serve to detect missing and double observations (Step 3) and can be used to model temporal dynamics (e.g., fluctuations across weekdays). Table 1 illustrates some dataset rows after applying temporal synchronisation.

#### 2.3.3 Step 3. Data Cleaning

Prior to merging multiple data sources, a further *required* step concerns data cleaning, that is the identification and treatment of missing and inaccurate data, such as incomplete, double and careless responses. Incomplete responses can be identified by inspecting missing values. Missing data are common in ILD, for instance, due to technical issues or lack of compliance. Several procedures have been proposed to quantify and correct the statistical biases implied by missing data, but their discussion is beyond the scope of the present work (see Ji et al. 2018). Here, we removed all participants that did not respond to any ESM questionnaire (*n* = 36, 1.8%) and all partial responses (i.e., cases where only some of the considered variables were responded) (*n* = 331, 18%).

Double responses can be easily identified by using the time point identifiers created in Step 2. For instance, we inspected the dataset looking for cases with the same ‘ID’, ‘day’ and ‘beep’ value, but no double responses were detected. In other cases, double responses should be removed based on their submission order (i.e., if timestamps are available, we recommend to only keep the first response) or other criteria (e.g., in case of incomplete responses, we might keep the response with less missing scores).

When self‐report data are collected, further procedures are *recommended* to identify and remove careless responses, i.e., cases of participants answering with insufficient effort/attention to be identified through attention‐check items (e.g., “*Answer this question with the value 2*”) or post hoc statistics (e.g., long‐string analysis) (Curran 2016). As a simple illustration, we just removed one case with extremely fast response times (i.e., less than 1.5 s per item), while we refer to Curran (2016) and Eisele et al. (2022) for more extensive suggestions. Further potential biases implied by self‐report ILD measures concern the tendency to respond more extremely in the first than in subsequent measurement occasions, so that initial responses might be considered as ‘calibrations’ and removed from the analyses (Shrout et al. 2018).

Finally, once the dataset has been cleaned from incomplete and inaccurate responses, it is *recommended* to consider filtering participants based on their compliance rate, i.e., percentage of valid data points over the number of scheduled measurements. As in the steps above, there is no definitive agreement on compliance rate thresholds, with suggested cut‐offs from 30% to 75% (e.g., see Kirtley et al. 2021). Compliance thresholds should also be considered depending on the focus of the target analyses. For instance, since our example focuses on within‐day variability, we removed all participants with less than two valid observations per day (*n* = 51, 29.1%).

Overall, the applied set of data‐cleaning procedures led to the exclusion of 90 participants (42.6%) and 637 data points (31.6%).

#### 2.3.4 Step 4. Data Merging

**FIGURE 1:**
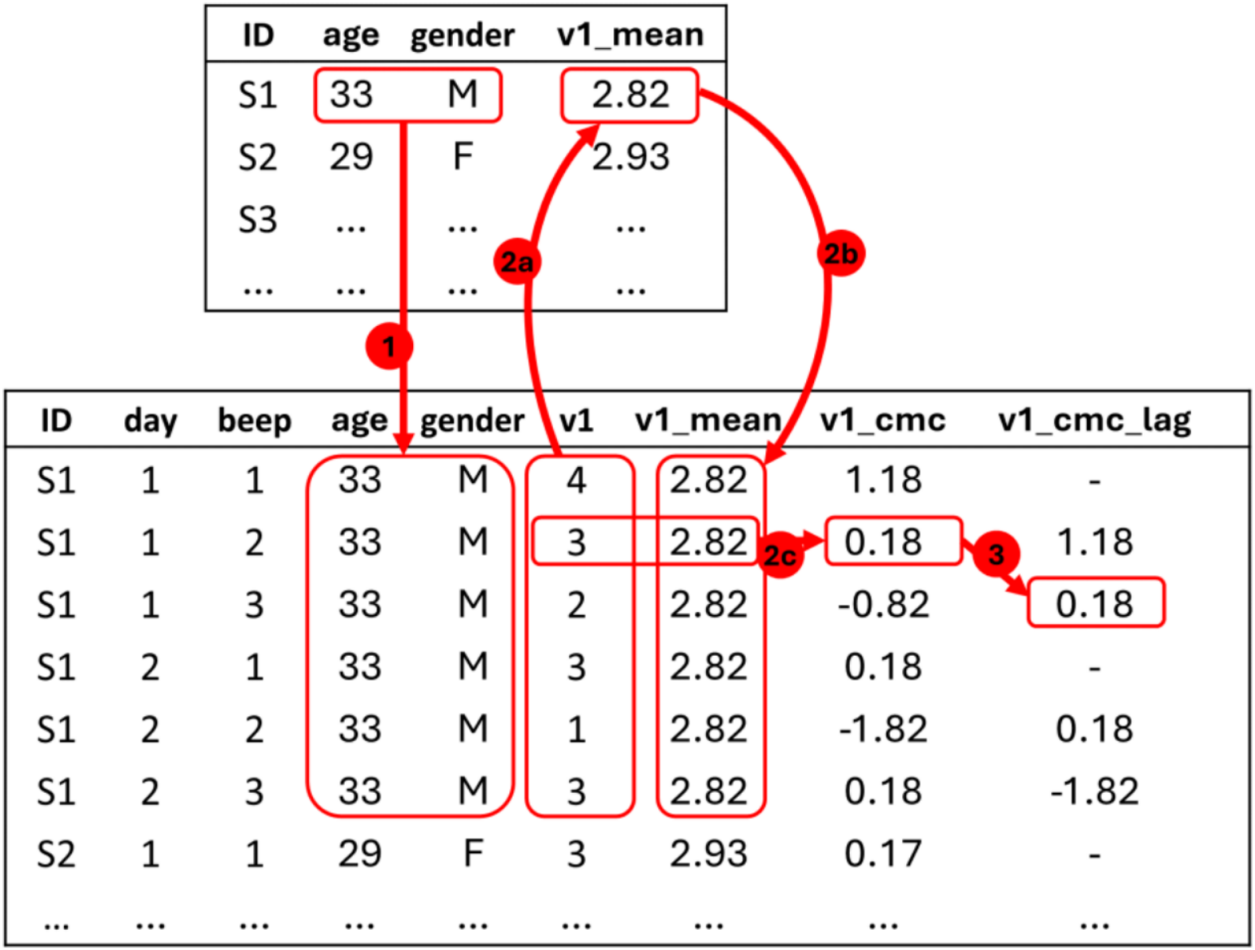
Visualisation of data merging (1), centering (2a = cluster mean computation; 2b = cluster mean data merging; 2c = cluster‐mean‐centering) and lagging (3) across the wide (top panel) and the long‐form datasets (bottom panel).

ILD often involve multiple data sources, such as the preliminary and ESM questionnaires included in our example. The use of multiple data sources *requires* merging the corresponding datasets into a unified dataset based on shared columns such as our ‘ID’, ‘day’ and ‘beep’ identifiers. Figure 1 (arrow 1) shows how participants' age and gender data from the preliminary questionnaire dataset can be merged with ESM ratings by aligning different sampling rates (i.e., one vs. up to six observations per participant) through shared columns (i.e., the ‘ID’ column). In this context, the first dataset of time‐invariant variables is referred to as the *wide‐form dataset*, whereas the second one is the *long‐form dataset*, where time‐invariant variable values (‘ID’, age and gender) repeat identically over all rows associated with the same participant (see Figure 1).

#### 2.3.5 Step 5. Data Centering

The wide‐ and long‐form datasets are needed to decompose time‐varying variables into their within‐ (level‐1) and between‐individual components (level‐2) through data centering, which is *required* for descriptive and regression analyses. Moreover, when multi‐item self‐reports are collected, data centering can also be applied to raw item scores to evaluate psychometric properties (Step 6). Time‐varying variables can be centered in multiple ways (Hamaker and Grasman 2015), but the most common one is to subtract the mean variable value for each participant (i.e., cluster‐mean‐centering). This is exemplified in Figure 1 for a negative valence item (‘v1’). First, we computed the mean item score for each participant (cluster mean). Second, we added such a newly created variable (‘v1_mean’) to the long‐form dataset in a way that the same value identically repeats over all rows associated with the same participant. Finally, we computed the cluster‐mean‐centered variable (‘v1_cmc’) by subtracting the corresponding cluster mean from the original variable (i.e., ‘v1’—‘v1_mean’). While ‘v1_mean’ quantifies the between‐individual component of ‘v1’, ‘v1_cmc’ indexes its within‐individual variability. In other words, the former represents stable individual differences in ‘v1’ scores, whereas ‘v1_cmc’ stands for score fluctuations over time. Positive ‘v1_cmc’ values indicate occasions where ‘v1’ scores were higher than the participant's average, negative values indicate lower‐than‐average scores and zero values indicate average scores for a given participant.

#### 2.3.6 Step 6. Psychometrics

Psychometric evaluations are *required* in any case where quantitative self‐reports are considered. While psychometrics can be considered a proper data analysis step, they can also reveal insights into data quality, prompting the need for additional data manipulation. At a lower level (*required*), item score distributions should be inspected, and inter‐item correlations should be separately computed for cluster‐mean (level‐2) and cluster‐mean‐centered (level‐1) scores (see Revelle and Wilt 2019). This allows for verifying the response range and distribution for each item, ascertaining that item scores from the same scale are positively correlated at both levels and identifying scores to be reversed.

At an intermediate level (still *required*), reliability indices can be computed based on variance decomposition. While the reliability of time‐invariant scales can be evaluated using standard methods (e.g., Cronbach's α), ESM imply more complex procedures. Specifically, we suggest computing the *between‐individual reliability index* (R_kF_), quantifying the scaleability to reliably discriminate different individuals and the *sensitivity‐to‐change index* (R_C_), informing on the scaleability to reliably detect systematic within‐individual changes over time (Revelle and Wilt 2019). Other indices such as the intraclass correlation coefficient (ICC) should be used with single‐item measures. Particularly, the ICC is a widely used statistic to quantify the proportion of variance attributable to between‐individual differences relative to the total variance. In ILD, this measure is often interpreted as an indicator of intra‐individual stability over time, as it reflects the extent to which an individual's scores on a specific variable remain consistent across repeated measures (e.g., Perkinson‐Gloor et al. 2015).

At a higher level of detail, it is *recommended* to run multilevel confirmatory factor analyses (MCFA) (Kim et al. 2016) for evaluating the hypothesised measurement models at both levels. It is worth mentioning that MCFA also allows testing cross‐level invariance (i.e., invariance of factor loadings across levels), which is a fundamental requirement for multilevel constructs conceptualised with the same meaning at both levels (e.g., level‐2 job control is the aggregate version of level‐1 task control, rather than being a different construct) (Steegen et al. 2016). Moreover, MCFA allows estimating further level‐specific reliability coefficients such as McDonald's ω (Geldhof et al. 2014). For instance, in our case, the three variables showed satisfactory reliability with ω_within_ ranging from 0.73 to 0.83 and ω_between_ ranging from 0.91 to 0.95.

Psychometric analyses and the associated results are better depicted at https://osf.io/sxh4q/, whereas we refer to Menghini et al. (2023) for more exhaustive evaluations of our considered measures.

#### 2.3.7 Step 7. Composite Indicators

Once psychometric qualities have been verified, it is *required* to aggregate raw item scores into composite scores (unless latent‐variable models are planned). With self‐reports, this is usually done by averaging or summing item scores to get a single observed variable for each construct (Weermeijer et al. 2022). With other types of data (e.g., physiological), composite indicators might be needed as well, for instance, to aggregate multiple data points over lower time periods (e.g., hourly average heart rate). In any case, descriptive and inferential analyses *require* decomposing each composite indicator into its within‐ and between‐individual components, following the same procedures described in Step 5. Here, we obtained a total of nine variables, namely the composite score of negative valence, task demands and task control, and the corresponding cluster‐mean and cluster‐mean‐centered scores.

#### 2.3.8 Step 8. Lagging and Leading

A final step that is *required* when data are planned to be analysed with autoregressive or other time‐lagged models (e.g., Hamaker and Grasman 2015) involves data lagging and leading. For instance, Figure 1 illustrates the lagging of ‘v1_cmc’, with each value being moved one row forward so that each ‘v1_cmc_lag’ value corresponds to the ‘v1_cmc’ value obtained in the previous time point within the same day and participant. Data leading implies the opposite transformation (i.e., moving values one row backward) and it is exemplified at https://osf.io/sxh4q/. Notably, both procedures imply a loss of data, namely the exclusion of the first daily value from the lagged variable (i.e., because there is not a preceding value to be moved forward) and the last value in the case of data leading.

Table 2 Summarises the data manipulation steps introduced above.

**TABLE 2:** Summary of the required and recommended data manipulation checks.

Data manipulation step	Required	Recommended
1. Data reading	Read data files Merge data files (when multiple files are exported from the same data source) Retain participant identifier, temporal coordinates and core study variables	Remove unused columns Rename variables strategically
2. Temporal synchronisation	Verify that temporal coordinates are correctly recorded Align time format across data sources	Create time point identifiers
3. Data cleaning	Remove/flag incomplete responses Remove double responses	Remove/flag careless responses and respondents Remove/flag the first data points (initial elevation bias) Remove/flag participants with low compliance rates
4. Data merging	Merge multiple datasets into a unified dataset (when multiple data sources are considered)	
5. Data centering	Compute the cluster‐mean and the cluster‐mean‐centered version of each time‐varying quantitative variable	
6. Psychometrics	Plot and compute level‐specific correlations among item scores Compute level‐specific reliability indices R_kF_ and R_C_ (when multi‐item self‐reports are used) Compute ICC for each time‐varying quantitative variable	Conduct MCFA and test cross‐level invariance (when multi‐item self‐reports are used) Compute level‐specific McDonald's ω
7. Composite indicators	Aggregate scores for each variable Compute cluster‐mean and cluster‐mean‐centered versions of each composite score	
8. Lagging and leading	Move forward/backward those variables to be related to other variables measured in previous/ following occasions	

Abbreviations: ICC, intraclass correlation coefficient; MCFA, multilevel confirmatory factor analysis; R_C_, sensitivity‐to‐change index; R_kF_, between‐individual reliability index.

## 3 Data Analysis

Following data pre‐processing, we computed descriptive statistics, zero‐order correlations at both within‐ and between‐individual levels (i.e., by correlating cluster‐mean‐centered values from the long‐form dataset and cluster‐mean values from the wide‐form dataset, respectively) and the ICC for each time‐varying variable. Then, we fitted two interactive models investigating JDC hypotheses. In Model 1, both task demands and task control were included as level‐1 predictors (i.e., cluster‐mean‐centered composite scores) and we tested their within‐individual interaction, whereas Model 2 included the cross‐level interaction between cluster‐mean‐centered task demands (level 1) and the cluster means of task control (level 2). Both models included age and gender as covariates and were fitted with R 4.4.1 (R Core Team 2024).

## 4 Results

Descriptive statistics are shown in Table 3. ICCs indicate balanced variability across levels for each time‐varying variable, with slightly greater level‐1 variability. Correlations were in the expected directions at both levels, with negative valence correlating positively with task demands and negatively with task control. At level 2, participants' age correlated positively with task demands and negatively with task control, whereas it was almost uncorrelated with negative valence.

**TABLE 3:** Descriptive statistics.

			Correlations		
Variable	Mean (SD)/ Frequency	ICC	1.	2.	3.
1. Negative valence (1–7)	3.31 (1.12)	0.46		0.14***	−0.22***
2. Task demand (1–7)	4.00 (1.34)	0.43	0.25**		−0.12***
3. Task control (1–7)	4.19 (1.51)	0.41	−0.40***	−0.09	
4. Age (years)	35.56 (9.87)	—	0.05	0.13	−0.21*
5. Gender	60 F (49.59%)	—	—	—	—

*Note:* Within‐individual correlations are shown above the main diagonal; between‐individual correlations are shown below. No. observations = 1,378, No. of participants = 121. *p < 0.05; **p < 0.01; ***p < 0.001.

Abbreviations: ICC, intraclass correlation coefficient; SD, standard deviation.

**FIGURE 2:**
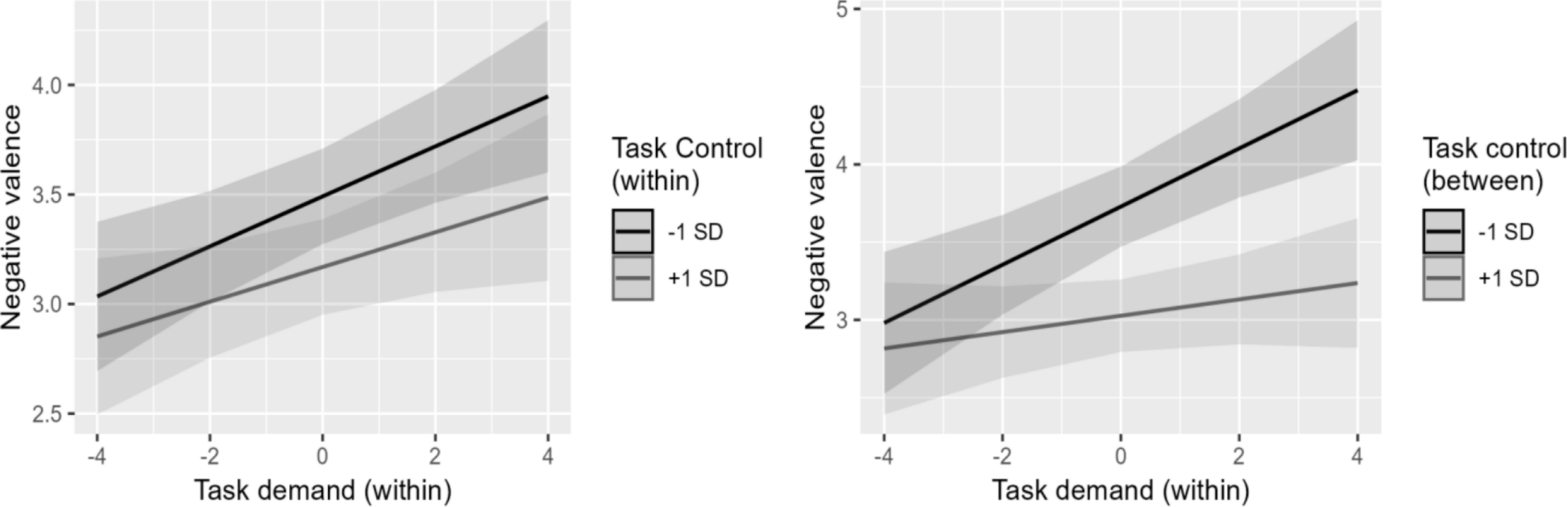
Estimated interactions between task demand and task control at the within‐ and between‐individual level.

Regression outputs are shown in Table 4. In both models, negative valence was positively associated with task demands and negatively associated with task control, consistent with the strain hypothesis. In contrast, a substantial interaction was only found in Model 2, where a stronger within‐individual relationship between level‐1 task demands and negative valence was predicted for individuals with lower average task control (see Figure 2).

**TABLE 4:** Regression models testing JDC hypotheses.

Predictors	Model 1 B (SE)	Model 2 B (SE)
(Intercept)	3.20 (0.29)***	4.80 (0.43)***
TDw (1–7)	0.10 (0.03)**	0.37 (0.13)**
TCw (1–7)	−0.15 (0.02)***	
TCb (1–7)	—	−0.32 (0.07)***
Age (years)	0.00 (0.01)	0.00 (0.01)
Gender [M]	−0.02 (0.15)	−0.06 (0.14)
TDw × TCw	−0.02 (0.02)	—
TDw × TCb	—	−0.06 (0.03)*
Random Effects		
τ^2^_00_	0.59	0.49
τ^2^_11_	0.04_TDw_	0.05_TDw_
ρ_01_	0.06	−0.03
σ^2^	0.59	0.61
Marginal/Conditional *R* ^2^	0.03/0.53	0.09/0.52

*Note:* *, *p* < 0.05; **, *p* < 0.01; ***, *p* < 0.001. No. of observations = 1,378, No. of participants = 121.

Abbreviations: SE, standard error; TCb, level‐2 task control; TCw, level‐1 task control; TDw, level‐1 task demands; ρ_01_, covariance between random intercept and random slope; σ^2^, residual variance; τ^2^_00_, random intercept; τ^2^_11_, random slope for the effect of task demand.

## 5 Discussion

The present work aimed at providing introductory and accessible guidance and code for implementing what we consider the most critical data manipulation steps preparing ILD data for subsequent analyses. This was done by specifically targeting applied psychologists, who are increasingly using ILD to answer specific research questions, conduct ecological momentary assessments and deliver interventions. Here, we highlighted key required and recommended procedures, including data reading, cleaning, merging of multiple data sources, data centering, lagging and leading, up to psychometric assessments and computation of composite indicators. The attached R code was designed to be as simple as possible, while we provided detailed comments and references to more introductory materials and advanced resources.

Our work emphasises the importance of transparently reporting pre‐processing procedures. While providing open data and materials is always recommended to improve the reproducibility of a study, we also highlight a range of alternative ways to manipulate ILD data, with each step involving multiple degrees of freedom. Due to the higher complexity of ILD compared to more common designs, resulting in a larger number of data pre‐processing choices, it is particularly critical to justify and possibly pre‐register the decisions taken at each step (see Kirtley et al. 2021).

Moreover, considering the lack of agreement in many data manipulation steps (e.g., handling of missing data, thresholds for compliance rate, etc.), it is also highly recommended to check the robustness of the findings by verifying that they are not implied by specific arbitrary choices (e.g., the specific compliance cut‐off used to exclude participants). That is, rather than focusing on a single path of data manipulation, it is suggested to pre‐process and analyse the data over a range of reasonable ways and discuss the robustness of the findings across the considered forking paths. Such a *multiverse approach* is increasingly used in many fields of psychology research (e.g., Calignano et al. 2023; Steegen et al. 2016), and it was recently applied by Weermeijer et al. (2022) to the pre‐processing of ESM data. By manipulating the compliance criterion of exclusion from 0% to 50%, the inclusion vs. exclusion of the first day of data collection and the computation of composite scores as means, medians or modes, the authors highlighted the potential impact of pre‐processing choices on the study conclusions. To illustrate such a multiverse approach to ILD data manipulation, we integrated our R code into a unified function allowing us to set the main options implied by each step, and we illustrated the robustness of our results to a range of alternative scenarios (see https://osf.io/sxh4q/).

Finally, our work provides some substantive insights into the applicability of the JDC model (Karasek 1979) at the within‐individual level. By repeatedly administering task‐level measures of job demands and control to analyse their direct and interactive relationships with mood (i.e., negative valence), we replicated previous findings supporting the strain (i.e., main effect) but not the buffer hypothesis (i.e., interaction) within individuals over time (Johnston et al. 2016; Totterdell et al. 2006). In other words, we found more negative moods after job tasks characterised by higher‐than‐usual demands and lower‐than‐usual control, whereas experiencing higher control than usual did not substantially buffer the momentary effect of task demands. Moving a step forward, we also analysed the cross‐level interaction between the two predictors, highlighting a weaker within‐individual relationship between task demands and negative valence for those workers reporting higher average control levels, in line with the buffer hypothesis and the idea that job control might exert more stable effects than job demands (Ilies et al. 2010).

Our tutorial is far from being comprehensive, but we hope it provides a starting point for applied psychologists approaching ILD for the first time. While we addressed the lack of guidance on ILD data manipulation, we are also aware of recent promising developments such as the ESM data pre‐processing framework by Revol et al. (2024), exhaustively covering ESM data manipulation steps and providing detailed R syntax as well as a dedicated R package that we recommend to expert readers. Moreover, we did not provide in‐depth guidance on relevant procedures such as handling of missing data and detection of careless respondents. Finally, while we focused on multilevel models due to their wide usage in applied psychology, we did not detail further statistical techniques such as dynamic structural equation modelling (DSEM), which might require some deviations from the procedures described in this work (see Zhou et al. 2021).

## 6 Conclusion

In recent years, there has been a growing interest in ILD in applied psychology, particularly within the organisational space. Despite this growing interest, handling ILD data poses considerable challenges also due to the absence of introductory guidelines. Our tutorial addresses this gap by providing a foundational guide on ILD data manipulation. As the use of ILD in applied psychology keeps growing, establishing robust practices in data pre‐processing becomes relevant for the advancement of open science practices and reproducibility principles in the field. Hence, we hope that our guide will contribute to fostering a more accessible and efficient approach to handling ILD data in applied psychology.

## Ethics Statement

All procedures performed in studies involving human participants were in accordance with the ethical standards of the institutional research committee of the departments of psychology at the University of Padova (protocol 2760) and with the 1964 Helsinki Declaration and its later amendments or comparable ethical standards.

## Consent

Informed consent was obtained from all participants included in the study (see Menghini et al. (2023)).

## Conflicts of Interest

The authors declare no conflicts of interest.

## Data Availability

The R code used to implement the data manipulation steps highlighted in the manuscript and to analyse the pre‐processed data are available from the anonymized public repository linked at: https://osf.io/sxh4q/?view_only=b74d2e84b5b04017b46e578570058e76.

## Endnotes

^1^
Back‐office workers primarily engage in administrative, support or operational tasks that do not involve direct interaction with clients or customers, such as data management and document processing.

^2^
The choice of sampling three non‐consecutive workdays was justified by the aim to minimise participants' efforts while maximising the variability of the measured variables over different workdays (see Menghini et al. (2023)).
